# New Azo Derivatives of Ethanol Lignin: Synthesis, Structure, and Photosensitive Properties

**DOI:** 10.3390/ma16041525

**Published:** 2023-02-11

**Authors:** Valentina S. Borovkova, Yuriy N. Malyar, Natalia Yu. Vasilieva, Andrey M. Skripnikov, Vladislav A. Ionin, Valentin V. Sychev, Viktor A. Golubkov, Oxana P. Taran

**Affiliations:** 1 Institute of Chemistry and Chemical Technology, Krasnoyarsk Science Center, Siberian Branch, Russian Academy of Sciences, Akademgorodok 50/24, 660036 Krasnoyarsk, Russia; 2 School of Non-Ferrous Metals and Materials Science, Siberian Federal University, pr. Svobodny 79, 660041 Krasnoyarsk, Russia

**Keywords:** lignin, diazonium compounds, azo lignins, cis–trans isomerism, photosensitivity

## Abstract

Water-soluble azo derivatives of lignin were synthesized by the azo coupling reaction using organosolv ethanol lignin and diazonium salts based on sulfanilic acid and p-nitroaniline. The structure of azo derivatives of lignin were studied by nuclear magnetic resonance, Fourier-transform infrared spectroscopy, and gel permeation chromatography. It was found that the azobenzene bonds formed in the azo coupling reaction of macromolecules impart the photosensitive properties to the synthesized polymers via cis–trans photoisomerization of the diazobenzene group. It was shown experimentally that the synthesized polymers exhibited good solubility both in the aqueous media in a wide (2–12) pH range and in DMSO and THF organic solvents, which opens up new prospects for their application.

## 1. Introduction

The continuous striving towards mastering new directions in the pulp and paper and hydrolysis industries has made biomass the leading raw material for the sustainable development of quite a few promising areas [1,2,3,4]. The lignocellulosic materials, owing to their high-value characteristics, abundance in nature, renewability, availability, and ease of production, can contribute to the rapid growth of the transition from fossil to renewable carbon resources [5,6]. In contrast to conventional fossil feedstocks, which are mostly hydrocarbons, lignocellulosic biomass is a functionalized biopolymer composed of three main biopolymers: cellulose, hemicellulose, and lignin.

Lignin is one of the richest multifunctional aromatic biomass resources [7]; it contains many aliphatic and phenolic OH groups and stilbene and arylenol ether groups offering vast opportunities for functionalization [8,9,10]. However, the global pulp and paper industry and bioethanol processing enterprises process 98% of lignin as low-value fuel or waste [10]; in other words, lignin is not used to its maximum potential.

To expand the use of lignin resources, new methods for modifying lignin and possible uses of the polymers based on it are being intensively sought. The introduction of different chemicals into the structure of polymers imparts new physicochemical properties, which can be controlled by external factors, e.g., light, heat, and pH [11,12]. In particular, esterification of the phenolic hydroxyl groups of lignin with anhydrides or acid halides of long-chain aliphatic acids can turn lignin from brittle to elastic [13]. There are quite a few examples of modifying the lignin properties by the organic synthesis methods [14,15]. A reaction that is well-known in organic chemistry and has been developed comprehensively with respect to lignin is the azo coupling reaction.

Azo polymers containing azochromophores have attracted much attention due to the unique trans–cis isomerization properties of the azochromophores when exposed to ultraviolet (UV) or visible light and have broad prospects for molecular electronics such as optical storage devices and optical switches [11,16,17] and for cosmetology [18].

In addition, there are studies [19] reporting the results of laboratory testing of modified lignins (azo derivatives of lignin) obtained in the reaction of sulfate lignin with diazonium salts as inhibitors of undesired thermopolymerization of reactive unsaturated compounds during processing of pyrocondensates. They exhibit good inhibitory properties similar to those of the well-known polymerization inhibitor 2,6-ditert-butyl-p-cresol (Ionol). In view of this, the production and use of various environmentally sensitive lignin-based polymers are important for biomass utilization.

A promising lignin-containing raw material for investigations is the Siberian fir (*Abies Sibirica Ledeb.*) that is widely distributed over the northeastern regions of the European part of Russia and in Western and Eastern Siberia [20]. For many years, this evergreen coniferous tree [20] has been used in various fields, in particular, in medicine for the prevention and treatment of diseases [21].

The aim of this study was to develop the modification methods based on introducing azobenzene groups into terminal units of lignin from the *Abies Sibirica Ledeb.* wood, to investigate the functionalization efficiency by nuclear magnetic resonance (NMR), Fourier-transform infrared (FTIR) spectroscopy, and gel permeation chromatography (GPC), and to estimate the photosensitivity of modified lignins in the UV and visible spectral ranges.

## 2. Materials and Methods

For the synthesis and physicochemical study of azo derivatives of organosolv lignin, the samples of ethanol lignin prepared from the *Abies Sibirica Ledeb.* wood by the original technique [22] and the samples sulfated with 1,4-dioxane and urea were used [23].

### 2.1. Fourier-Transform Infrared Spectroscopy

To identify the functional groups contained in the synthesized azo polymers, IR spectra in the wavelength range of 4000–400 cm^−1^ were recorded on a Tensor 27 FTIR spectrometer (Bruker Optik Gmbh, Ettingen, Germany) at the Krasnoyarsk Regional Center for Collective Use, Krasnoyarsk Scientific Center, Siberian Branch of the Russian Academy of Sciences. The spectral data were processed using the OPUS software package, version 5.5. The samples for the FTIR spectroscopy investigations were prepared in the form of tablets in a potassium bromide matrix. The preparation conditions (time of mixing with potassium bromide, tableting pressure, and evacuation time) were the same for all the samples. The substance concentration in the tablets was constant and amounted to 4 mg per 1000 mg of KBr.

### 2.2. Nuclear Magnetic Resonance

Two-dimensional (2D) nuclear magnetic resonance (NMR) spectra were recorded on a Bruker AVANCE III 600 spectrometer (Bruker BioSpin, Rheinstetten, Germany) at working frequencies of 600 MHz (^1^H) and 155 MHz (^13^C) at 25 °C using 5 mm ampoules. About 0.08 g of lignin was dissolved in 0.6 mL of deuterated dimethyl sulfoxide and then 2D NMR spectra were recorded in the heteronuclear single quantum correlation (HSQC) experiments. The spectral widths in the ^1^H and ^13^C measurements were 8000 and 28,000 Hz, respectively. The number of accumulations was 16 thousand with a delay of 5 s for ^1^H and 20 thousand with a delay of 7 s for ^13^C. The δC 40.1; δH 2.5 solvent signal was used as an internal standard. The cross-signals of the HSQC spectra were interpreted using the literature data.

### 2.3. Gel Permeation Chromatography

The average molecular weight *M*_w_, number average molecular weight *M*_n_, and the polydispersity index (PDI) of the azo lignins were determined by gel permeation chromatography (GPC) using an Agilent 1260 Infinity II Multi-Detector GPC/SEC System (Agilent Technologies, Santa Clara, CA, USA) with triple detection: refractometer (RI), viscometer (VS), and light scattering (LS). The separation was performed on two combined PL Aquagel-OH Mixed-M columns using the mixture 0.2 M NaNO_3_ + 0.01 M NaHPO_4_ as the mobile phase for the aqueous solutions and tetrahydrofuran (THF) as the mobile phase for the organic solutions. The columns were calibrated using polyethylene glycol (PEG) and polystyrene (PS) polydisperse standards (Agilent, Santa Clara, CA, USA). The eluent flow rate was 1 mL/min and the injected sample volume was 100 µL. Before the analysis, the water-soluble samples were dissolved in water (1.5 mg/mL) and the remaining samples, due to their insolubility in water, were dissolved in THF (1.5 mg/mL) and filtered through a 0.45 µm Millipore PTFE membrane filter. The data collection and processing were performed using the Agilent GPC/SEC MDS software version 2.2.

### 2.4. Thermal Analysis

The thermogravimetry study was carried out on a NETZSCH TG 209 F1 thermal analyzer (Netzsch, Selb, Germany) and the data obtained were analyzed. The thermal decomposition of the samples was analyzed in nitrogen in the temperature range from 25 to 700 °C. The protective and blowout gas flow rates were 20 mL/min. The samples were heated in cylindrical corundum crucibles in the dynamic temperature regime (10 °C/min). The TG 209 F1 analyzer was calibrated according to the instructions and using the reference manifestations that appear with the device The samples were weighed on an XFR-125E laboratory balance. The measurement data were processed using the NETZSCH Proteus Thermal Analysis 4.8.4 software supplied with the instrument.

### 2.5. Spectrophotometry Analysis

The photosensitivity of the free azo derivatives of lignin was examined on a SPEKOL-1300 spectrophotometer (Analytik Jena AG, Jena, Germany) in a quartz cuvette with an optical path length of 1 cm. The samples were dissolved in dimethyl sulfoxide (DMSO). The volume of the solution with a concentration of 3 ∙ 10^−3^ M was 200 μL. The degree of isomerization was monitored by the height of a peak at 190–500 nm. Prior to irradiation, the solution of the azo derivatives of lignin contained a mixture of the cis and trans isomers with a predominance of the latter. To convert the compound to the trans-configuration, the solution was irradiated by a UV lamp light with a wavelength of 470 nm for 1 min. To obtain the cis-isomer, the exposure at a wavelength of 365 nm lasted for 1 h. After that, the reverse isomerization at a wavelength of 470 nm for 2 min was performed.

## 3. Results

**Figure 1:**
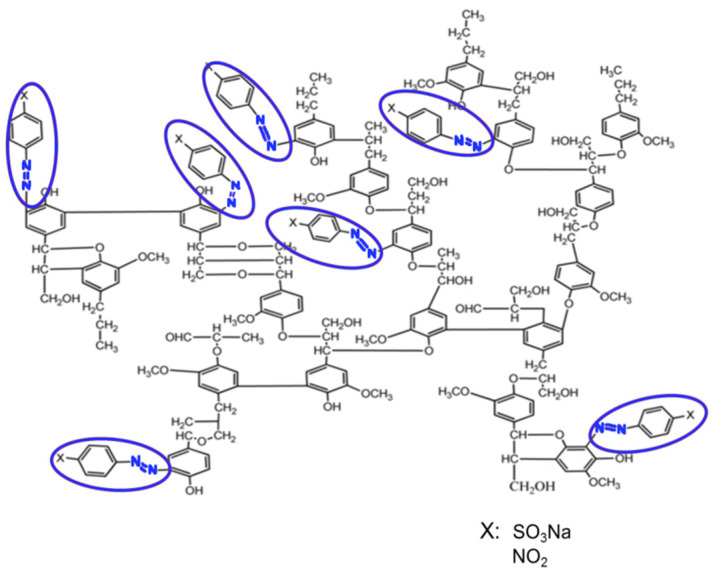
Positions of azo groups in the structure of *Abies Sibirica* lignin.

The azo derivatives were synthesized in the reaction of lignin with diazonium salts, which were obtained on the basis of sulfanilic acid and p-nitroaniline. It was shown that the interaction of lignin with diazo compounds induces the azo coupling reaction. The azo group becomes ortho to the phenolic hydroxyl. Figure 1 shows a representative structure of lignin of abies wood functionalized with p-nitroaniline and sulfanilic acid. Only 0.3–0.4 azo groups are attached to each free phenolic hydroxyl, which is related to the partial condensation of the ortho position (position 5) and/or partial esterification of phenolic hydroxyl. As was established previously, the azo group replaces hydrogen in the benzene ring only in the presence of free phenolic hydroxyl [24].

It is well known that, in the process of combining lignin with diazonium salts at a pH above 10, the diazo group is deactivated and turns into its inactive form, i.e., diazotate [25].

In addition, the strongly alkaline environment provokes a side arylation reaction, which adds nitrogen-free diazo radicals to lignin. This causes the release of gaseous nitrogen. Therefore, the synthesis was carried out in a weakly alkaline (about 8–10) medium.

### 3.1. Synthesis of Azo Lignin Using p-Nitroaniline

In the synthesis, 1.125 mL of water, 1.125 mL of concentrated hydrochloric acid, and 0.5 g of p-nitroaniline were put into a 100 mL reaction beaker. The solution was cooled to 0 °C in a mixture of ice and salt and a solution of 0.35 g of sodium nitrite in 1 mL of water (solution № 1) cooled to 0 °C was added to it.

**Figure 2:**
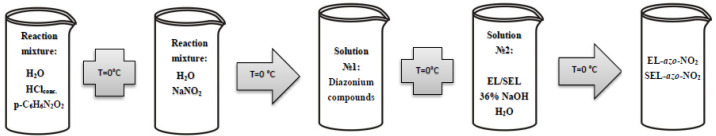
Scheme of lignin nitriding with p-nitroaniline.

In a separate beaker, 0.9 g of ethanol lignin (EL)/sulfated ethanol lignin (SEL) was mixed with 0.5 mL of the 36% sodium hydroxide solution and 1.5 mL of water (solution № 2). The resulting solution was cooled down in a mixture of ice and salt to 0 °C and the diazonium salt solution was gradually added to it under stirring. The reaction mixture was left for 0.5 h in an ice bath and then the precipitate was filtered off on a Buchner funnel. Next, the obtained azo lignin (EL-*azo*-NO_2_/SEL-*azo*-NO_2_) was dried in air. The process scheme is shown in Figure 2.

### 3.2. Synthesis of Azo Lignin with Sulfanilic Acid

Solution № 1 was prepared from 1 g of sulfanilic acid and 2.5 mL of 2 N sodium hydroxide solution. A solution of 0.4 g of sodium nitrite in 5 mL of water was added to the alkaline solution. After cooling down the resulting solution to 0–5 °C, it was gradually poured into 10 mL of 2 N hydrochloric acid cooled to 5 °C under stirring.

In a separate beaker, 0.9 g of EL/SEL was dissolved upon heating in 5 mL of 2 N sodium hydroxide solution (solution № 2). The solution was cooled in a bath filled with ice.

**Figure 3:**
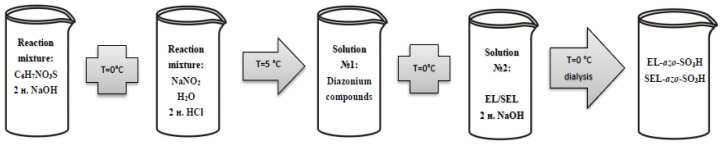
Scheme of nitriding lignin with sulfanilic acid.

After the completion of the reaction, cooled solution № 2 was rapidly added to obtained solution № 1 under stirring, which continued for 0.5 h. To remove unreacted substances, the product was dialyzed in an MF-503-46 MFPI dialysis bag (US) with a pore size of 3.5 kDa against water for 8–10 h; the water was changed every hour. After dialysis, the solution was evaporated to dryness under vacuum on a rotary evaporator and the solid water-soluble residue was obtained: azo lignin (EL-*azo*-SO_3_H/SEL-*azo*-SO_3_H). The process scheme is shown in Figure 3.

### 3.3. FTIR Spectroscopy Study of Azo Lignins

**Figure 4:**
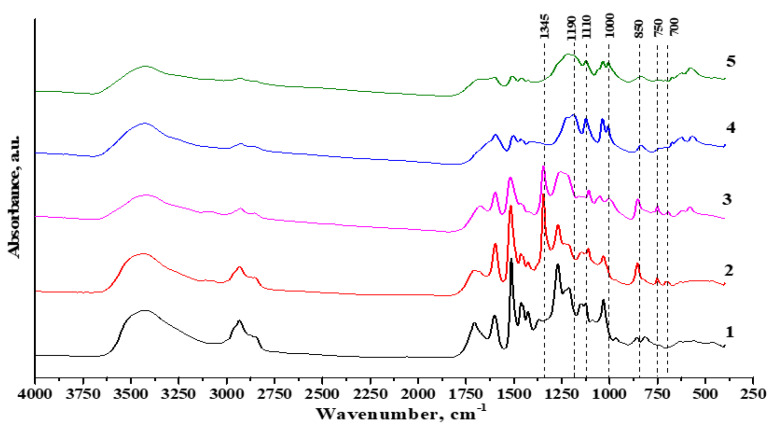
FTIR spectra of (**1**) EL, (**2**) EL-azo-NO_2_, (**3**) SEL-azo-NO_2_, (**4**) EL-azo-SO_3_H, (**5**) SEL-azo-SO_3_H.

When comparing the FTIR spectra of the initial lignin sample with its azo derivatives (Figure 4), some distinctive changes were found. In particular, the shape of the absorption band between 3100 and 3600 cm^−1^ corresponding to the OH group changed, apparently due to the conversion of phenolic OH groups to aliphatic ones [26].

The analysis of the FTIR spectra of the EL-*azo*-NO_2_ and SEL-*azo*-NO_2_ lignins revealed pronounced absorption bands with maxima at ~1500 and ~1345 cm^−1^ related to the symmetrical and asymmetrical stretching vibrations of the NO_2_ group associated with the aryl radical [27], which indicates the introduction of p-nitroaniline into the lignin system. The absorption bands with medium intensities at 700, 750, and 850 cm^−1^ are related to the NO_2_ vibrations as well [28].

In the EL-*azo*-SO_3_H and SEL-*azo*-SO_3_H FTIR spectra, the following changes were found: the appearance of absorption bands with maxima at 850 and 1110 cm^−1^ corresponding to the R–SO_2_–OH and R–SO–OH stretching vibrations, noticeable absorption bands around 1000 cm^−1^ corresponding to the S=O stretching vibrations, and the absorption band with a maximum at ~1190 cm^−1^ characterizing the C=S stretching vibrations.

These pronounced changes illustrate the efficient azo coupling reaction in the lignin samples.

### 3.4. Nuclear Magnetic Resonance Study of Azo Lignins

**Figure 5:**
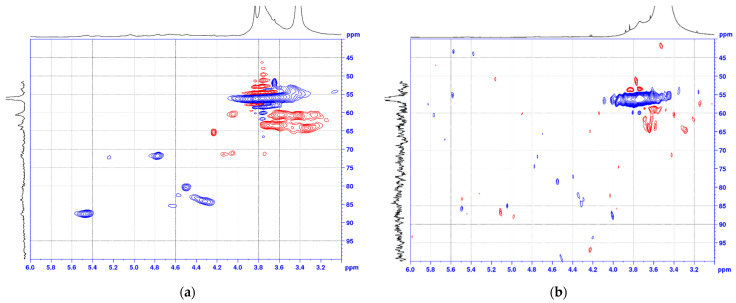
Aliphatic region of the 2D HSQC NMR spectra for (**a**) EL and (**b**) EL-*azo*-NO_2_.

**Figure 6:**
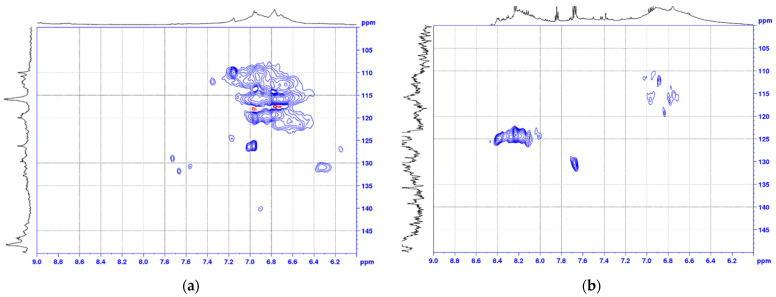
Aromatic region of the 2D HSQC NMR spectra for (**a**) EL and (**b**) EL-*azo*-NO_2_.

The comparison of the signals areas in the EL and EL-*azo*-NO_2_ [^1^H–^13^C] HSQC spectra showed that the NMR spectra of these samples were largely identical, but with some obvious changes (Figure 5a,b and Figure 6a,b).

The most intense signals in the aromatic region of the HSQC spectra (δ^13^C/δ^1^H 107-123/7.3-6.4) correspond to aromatic guaiacyl (G) structural units. The peaks assigned to the CH groups at positions 2, 5, and 6 of the guaiacyl ring in the initial and azo ethanol lignin are almost identical, which confirms the absence of nitriding over phenolic hydroxyls.

The signals of pinoresinol (D) fragments were identified in the spectra of both ethanol lignin and its azo derivative, except for D_γ_. The correlation signals of the pinoresinol fragments of abies ethanol lignin and azo ethanol lignin were similar.

Abies ethanol lignin and azo ethanol lignin contain phenylcoumarane fragments (the δ^13^C/δ^1^H ratios are 88.6/5.45 and 86.9/5.50, 52.8/3.61 and 53.0/3.59, 63.5/3.65 and 62.3/3.71, respectively), the peaks of which were identified in the HSQC spectra in the α, β, and γ positions. The pronounced signals at δ^13^C/δ^1^H = 56.1/3.76 and 56.1/3.73 for ethanol lignin and azo ethanol lignin, respectively, are indicative of a great number of methoxyl groups (OMe), which are important structural units of all lignins [28].

The comparison of the correlation signals from atoms in the α, β, and γ positions of the β-aryl ether (β-O-4) in ethanol lignin and azo lignin structures showed a difference between the signals of the corresponding atoms in the HSQC spectra in all the indicated positions of these structures.

Concerning the pinoresinol signals, the HSQC spectra showed that the azo derivative of lignin lacks pinoresinol (β–β′) fragments in all the positions (α, β, and γ).

These indicators point out a clear change in the initial lignin structure caused by the introduction of azo groups.

### 3.5. Gel Permeation Chromatography Study of Azo Lignins

One of the most important characteristics of the chemical processing of polymers containing azo derivatives of ethanol lignin is their molecular weight.

**Figure 7:**
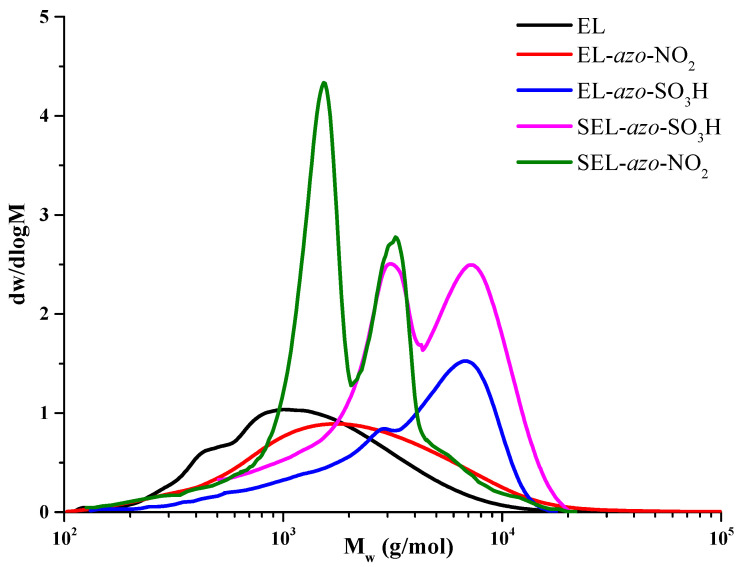
Molecular weight distribution curves for the abies lignin samples.

According to the data listed in Table 1, a trend towards an increase in the molecular weights (*M*_w_) of the azo derivatives can be expected, except for the SEL-*azo*-NO_2_ sample, in which the lignin structure is apparently partially destructed after introducing sulfo and azo groups into the system with the formation of a larger fraction of low-molecular-weight components, which affect also the molecular weight distribution profile (MWD) (Figure 7).

The comparison of the MWD profiles of the EL and EL-*azo*-NO_2_ samples analyzed in the THF medium revealed a shift of the curve to the higher molecular weight region as a result of modification of the initial lignin sample. As for the water-soluble azo derivatives of lignin, their modification with sulfanilic acid resulted in the formation of the EL-*azo*-SO_3_H product with a predominant content of macromolecular compounds.

The analysis of the azo and sulfo derivatives of lignin (SEL-*azo*-SO_3_H/SEL-*azo*-NO_2_) yielded a bimodal MWD with a pronounced peak around ~2000 g/mol, which corresponds to the nitriding products.

### 3.6. Thermal Analysis

**Figure 8:**
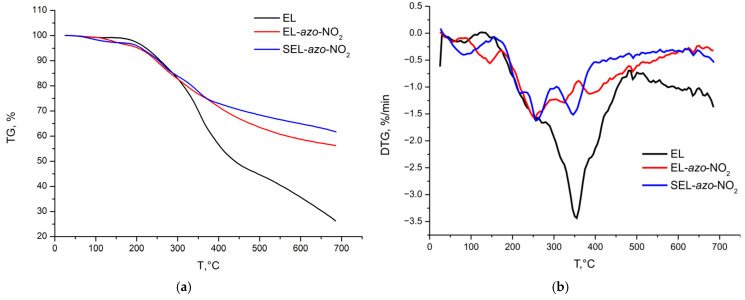

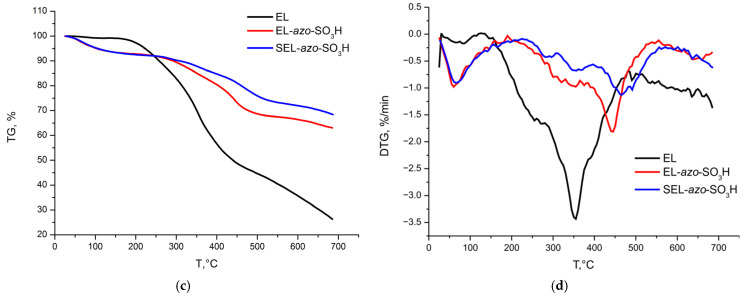
(**a**,**c**) TG and (**b**,**d**) DTG data.

The thermogravimetry (TG) and differential thermogravimetry (DTG) study was carried out on a synchronous thermal analyzer. The results obtained are presented in Figure 8.

It can be seen that the investigated lignins decomposed in a wide temperature range and this process finished mainly by 700 °C. The comparison of the thermolysis of the samples revealed different weight losses of the investigated lignins at the same temperatures. The native ethanol lignin sample exhibited the maximum weight loss up to the completion of pyrolysis. In the thermograms shown in Figure 8, the DTG curve of the initial lignin sample showed a broad peak typical of lignins with a shoulder between 200 and ~330 °C. In the temperature range of 230–260 °C, the lignin propanoic side chains degrade with the formation of methyl, ethyl, and vinyl derivatives of guaiacol. In addition, at temperatures of ≤310 °C, the ester bonds with the low chemical stability break [23]. The similar degradation processes occurred in the lignin samples modified with nitraniline. However, the main weight loss peak for modified ethanol lignin at 350 °C became noticeably lower, indicating the formation of thermostable condensed structures.

The thermograms of the EL-*azo*-SO_3_H and SEL-*azo*-SO_3_H samples also point out their elevated stability. After the first weight loss peak in the range of up to 100 °C, the samples lose their weight at a slower rate: the three minor peaks at 300, 360, and 450–500 °C are apparently related to the removal of sulfo groups [23].

### 3.7. Photochemical Study of Azo Lignins

The cis–trans photoisomerization reaction is a change in the configuration of a molecule during the transition from the initial stable ground state to the highly active excited state after absorption of UV light of a certain wavelength by the molecule. The molecule is transformed from a light-resistant to light-stable form. Such properties are typical, in particular, for a number of organic molecules containing this kind of isomers, including alkenes, alkynes, azo compounds, and aromatic compounds [29].

**Figure 9:**
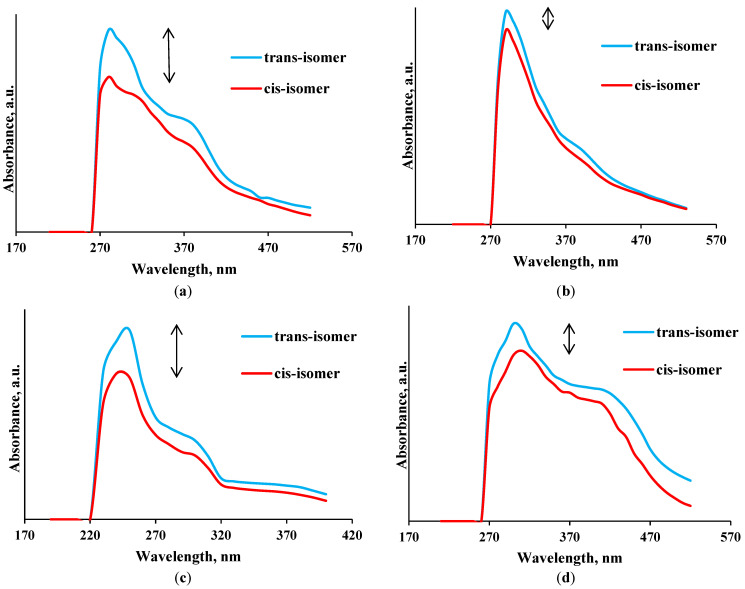
UV absorption spectra for (**a**) EL-*azo*-SO_3_H, (**b**) SEL-*azo*-SO_3_H, (**c**) EL-*azo*-NO_2_, (**d**) SEL-*azo*-NO_2_.

Since the obtained azo lignins contain a conjugated N=N double bond, they exhibit the photoisomerization phenomenon illustrated in Figure 9.

It can be seen that the photosensitive properties of the N=N chromophore group are determined by the reversible trans → cis → trans photoisomerization cycle. Importantly, the trans–cis isomerization occurs over a much longer time (1 h) than the cis–trans isomerization (1–2 min).

It addition, we would like to emphasize that the sulfated derivatives demonstrated a weaker photoisomerization effect. Obviously, this is due to the fact that the sulfate groups introduced originally in the lignin structure can replace hydrogen in aliphatic or aromatic OH group, thereby making steric obstacles for further attachment of the N=N group. Since sulfate groups can already occupy several positions in the aromatic ring and spatially prevent the addition of the N=N group, the subsequent insertion of diazo compounds can be sterically hindered, which affects directly the cis–trans isomerism results.

## 4. Conclusions

The modification methods based on the introduction of azobenzene groups into terminal units of *Abies Sibirica Ledeb*. lignin via the azo coupling reaction in the presence of p-nitroaniline and sulfanilic acid were proposed.

The functionalization efficiency was investigated by nuclear magnetic resonance and FTIR spectroscopy. It was found that the aromatic region of the HSQC spectra of azo ethanol lignin contains peaks indicative of the attachment of new functional groups to the γ position of the β-aryl ether and phenylcoumaran (δ^13^C/δ^1^H 123–126/8.0–8.4). It was found that, when analyzing the FTIR spectra, it is necessary to isolate the absorption bands with maxima at ~1500 and ~1345 cm^−1^ related to the symmetrical and asymmetrical stretching vibrations of the NO_2_ group, i.e., p-nitroaniline, as well as the absorption bands with maxima at 850 and 1110 cm^−1^ corresponding to the R–SO_2_–OH and R–SO–OH stretching vibrations occurring upon modification with sulfanilic acid.

It was established that lignin and its derivatives obtained in this study have different molecular weight distributions and weight average molecular weights (1617–4938 g/mol). The SEL-*azo* derivatives have lower molecular weights than azo lignins from the initial product due to the partial degradation and formation of low-molecular-weight sulfation products.

The examination of the photosensitive properties showed that the most pronounced cis–trans spatial changes occurred in the azo lignin samples obtained from initial ethanol lignin, which is preferred for future use.

## Figures and Tables

**Table 1:** Molecular weight characteristics of lignin and its derivatives.

Sample	*M*_n_ (g/mol)	*M*_w_ (g/mol)	PDI
EL	887	1854	2.09
EL-*azo*-NO_2_	1160	3445	2.97
EL-*azo*-SO_3_H	2843	4938	1.73
SEL-*azo*-SO_3_H	1262	2382	1.89
SEL-*azo*-NO_2_	807	1617	2.01

## Data Availability

All data generated during this study are included in the article.
